# Secondary Metabolism and Hormone Response Reveal the Molecular Mechanism of Triploid Mulberry (*Morus Alba L*.) Trees Against Drought

**DOI:** 10.3389/fpls.2021.720452

**Published:** 2021-10-06

**Authors:** Hui Liu, Hongmei Sun, Lijun Bao, Shuhua Han, Tian Hui, Rui Zhang, Minjuan Zhang, Chao Su, Yonghua Qian, Feng Jiao

**Affiliations:** ^1^The Sericultural and Silk Research Institute, College of Animal Science and Technology, Northwest A&F University, Xianyang, China; ^2^Shaanxi Key Laboratory of Sericulture, Ankang University, Ankang, China

**Keywords:** mulberry, drought stress, polyploidy, RNA-Seq, secondary metabolism

## Abstract

The improvement of a plant's tolerance to drought is a major endeavor in agriculture. Polyploid plants often exhibit enhanced stress tolerance relative to their diploid progenitor, but the matching stress tolerance is still little understood. Own-rooted stem cuttings of mulberry (Morus alba L.) cultivar Shinichinose (2n = 2x = 28) and Shaansang-305 (2n = 3x = 42) were used in this study, of which the latter (triploid) has more production and application purposes. The responses of triploid Shaansang-305 and diploid progenitor ShinIchinose under drought stress were compared through an investigation of their physiological traits, RNA-seq, and secondary metabolome analysis. The results showed that the triploid exhibited an augmented *abscisic acid (ABA)* content and a better stress tolerance phenotype under severe drought stress. Further, in the triploid plant some genes (*TSPO, NCED3*, and *LOC21398866*) and *ATG* gene related to ABA signaling showed significantly upregulated expression. Interestingly, the triploid accumulated higher levels of RWC and SOD activity, as well as more wax on the leaf surface, *but with less reductive flavonoid than in diploid*. Our results suggest triploid plants may better *adapt to* with drought events. Furthermore, the flavonoid metabolism involved in drought resistance identified here may be of great value to medicinal usage of mulberry. The findings presented here could have substantial implications for future studies of crop breeding.

## Introduction

Polyploidization has occurred numerous times throughout the evolutionary history of all plants and dramatically improved the successful adaptation of an extensive range of angiosperms to different habitats (Van de Peer et al., 2017; Chen et al., 2018; Wu et al., 2020). Polyploidy was involved in the speciation of many important crops and continues to play a vital role in the agricultural industry (Sattler et al., 2016). For example, polyploid potentiates tolerance to drought stress by regulating stomatal density and aperture size in tetraploidy cassava (Xiao et al., 2019a), consuming significantly less water under drought condition in tetraploid *Populus* trees (Hennig et al., 2015), and it could enhance the activity of enzymes related to antioxidant processes and sugar metabolism in trifoliate orange (Wei et al., 2019). Plenty of evidence on the molecular mechanism of polyploidy resistant to drought stress in various species has been reported. However, triploids Populus tremuloides aspen feature higher intrinsic water-use efficiency (iWUE) and incur greater potential water losses, so triploids may actually be less resilient to climate-induced stres*s* (Greer et al., 2018). Accordingly, the drought stress response of polyploidy plants relative to their diploid progenitor remains contentious. Moreover, many polyploidy studies have focused on allopolyploidy, which interferes with heterogeneous genome alongside its ploidy effects, meaning far less attention is paid to autopolyploid. Based on an admittedly limited number of examined species, autopolyploid may confer better stress tolerance to water deficit, such as in autopolyploid *Panicum virgatum L*., *Dianthus broter*i, and tetraploidy *Arabidopsis* (Del Pozo and Ramirez-Parra, 2014; Lopez-Jurado et al., 2019; Chen et al., 2020). However, there are relatively few such studies on woody plants, despite their unique and well-known physiological mechanisms for maintaining water balance, such as perennation, woody stem tissue formations, and long-distance transportation of water.

Genomic structural variation and enrichment of the genetic diversity caused by polyploidy, increasing in genetic variation, may significantly affect the morphology and physiology of the newly formed autopolyploids. Drought-resistant plants *exhibit* a strong water retention capability. Plants undergo two main forms of transpiration: stomatal transpiration and keratinous membrane transpiration. Stomatal transpiration is the major form of water consumption, whereas cuticular transpiration only accounts for 5–10% of transpired water in plants. Most studies focused on the changes in stomatal transpiration; however, there has been increasing interest in wax vis-à-vis drought stress resistance in recent years. Furthermore, leaves with a greater amount of leaf surface wax and larger crystal size and density displayed reduced leaf post-harvest water loss, which could be of value for the selective breeding of improved mulberry (Mamrutha et al., 2010a). In fact, hormones play a key role in tolerance of woody plants to drought and salt stress; both abscisic acid (ABA) and ethylene have regulatory functions for mulberry's tolerance to abiotic stress factors, and they are some key factors regulating stomatal function (Liu et al., 2019b; Shang et al., 2019). Secondary metabolites are strongly related to plant defense response signals (Zhao et al., 2005; Jwa et al., 2006; Pusztahelyi et al., 2015; Isah, 2019) and the stress-related genes and pathways associated with plant secondary metabolites (PSMs) biosynthesis in medicinal plants were explored by increasing transcriptomics and metabolomics analysis (Watson et al., 2015; Tuyiringire et al., 2018). Most evidence for whole genome duplications (WGD) promoting adaptation has been circumstantial, such as adapting to the changing environment. Chao et al. (2013) found that, naturally, autotetraploid have enhanced salinity tolerance through elevating potassium and reducing sodium levels. This can also be seen in polyploid plants (e.g. rice, Citrus grandis) that have an enhanced tolerance to drought and salt (Tu et al., 2014; Wei et al., 2019). In part, this is due to the polyploid affecting the gene expression programs associated with stress-related and hormonal pathways (Song et al., 2020). New phenotypes in the neopolyploid lineage facilitates the survival of polyploids in new environments, and subsequent adaptation results from increasing genetic variation which is beneficial for the development of ecosystems (Campbell et al., 2006). Compared to diploid plants, polyploid is commonly considered to have higher tolerance to more extensive ecological environments. However, the molecular mechanisms underlying physiologic changes liable for polyploid increased drought stress tolerance are still poorly understood.

Mulberry tree (*Morus alba* L.) leaves are the sole source of food for silkworms, and this woody plant is critical for breeding new cultivars that can adapt well to various environment conditions. In particular, mulberry is an anticipated bio-energy crop recognized as quite suitable for short rotation coppice forestry-based mitigation of rising CO_2_ levels, even in the face of intermittent drought events (Wu and Zhou, 2015; Slette et al., 2019). Mulberry also has considerable applied value in the pharmaceuticals industry (De Padua Lucio et al., 2018), and its growth in moderate soil moisture can change the content of secondary metabolites in leaves, many of which possess medicinal value. Various parts (root, stem or branch, leaf and fruit) of morus could be used in therapeutic drugs (Gryn-Rynko et al., 2016; Yin et al., 2017; Chen et al., 2021). Extracts of Morus alba has been applied to manage body weight, control appetite, and improve metabolic syndromes (Yimam et al., 2019). Hence, research on the tolerance to abiotic and biotic stresses in mulberry trees is essential for its appropriate variety selection so as to benefit not only sericulture but also the pharmaceuticals industry and the ecological environment. Triploids of mulberry cultivar often exhibit several excellent economic traits including high production of leaves, but the alterations in stress resistance caused by genetic dosage effect is unknown (Yan et al., 2019; Dai et al., 2020). It is necessary to evaluate the differential performance of plants which have different ploidy levels, and associated molecular mechanisms involved in drought resistance of mulberry trees, so as to provide a new way of improving stress tolerance capacity of mulberry and other similar woody plants.

The study provides important information on how ploidy levels are associated with drought resistance and how they may better cope with drought events. Furthermore, plants of roots induced by an asexual propagation genetic background were compared, avoiding the effect of grafted plants. In order to understand the molecular model of how mulberry trees resist drought stress, in this study we compared stress tolerance, transcriptome, and flavonoid metabolome between own-rooted stem cuttings of mulberry cultivar Shinichinose (2n = 2x = 28) and its autotriploid Shaansang-305 (2n = 3x = 42) (Jiao et al., 2020). The plant response variables investigated consisted of leaf water content, antioxidant activity, stress tolerance, gene expression levels, the secondary metabolite flavonoid, and epidermis wax content.

## Materials and Methods

### Plant Material and Drought-Stress Treatment

The cultivar autotriploid Shaansang-305 (2n = 3x = 42) were induced from the DNA duplicated cells in the shoot apical meristem of the diploid cultivar Shinichinose (2n = 2x = 28) treated by colchicine (Han et al., 2000; Jiao et al., 2020). Seedlings were planted in culture medium containing peat, vermiculite, and compost (v/v/v = 1:1:1). The experiment was carried out in a greenhouse under natural daylight conditions at the Northwest A&F University (Yangling, Shaanxi Province, China). Own-rooted stem cuttings of autotriploid Shaansang-305 and its parental diploid ShinIchinose were grown by tissue culture in this study. Drought stress was imposed on 3-month-old cutting plants. In the drought-stressed treatment, each pot had watering withheld initially, and when the soil water content dropped to ~ 30% field capacity, it was maintained at that level by weighing and watering back every day; the trial lasted 21 days. Control plants were grown with a normal water supply. Each treatment experiment was repeated three times. After the experiment, the leaves of plants of Day 0, Day5, Day 9, Day 15, and Day 21 samples were collected. Samples were collected in the morning (9–11 am). The three top-most fully expanded leaves from each plant were harvested for photosynthetic measurements, RNA extraction, and transcriptome sequencing. The middle part of the plant was used for physiological measurements and metabolomic assay. All samples were immediately stored in liquid nitrogen and kept at –80°C.

### Flow Cytometry Analysis

The ploidy levels of somatic cells in each sample was identified by flow cytometry analysis. Prior to the flow cytometry measurements, the young fresh leaves of apical were obtained and cleaved with razor blades in a nuclei extraction buffer and stained with propidium iodide (PI). The experiment was performed three times (independently); for each analysis a total of 10 000 nuclei were surveyed. Flow cytometry information and data were acquired using a FACS-calibur flow cytometer (BD Biosciences, San Diego, CA, USA).

### Microscopic Observation

Three disease-free mature plants with the same developmental stature (same number of leaves) were picked from each of the two cultivars. Leaves of each plant were removed for paraffin sectioning. This fresh tissue was placed into a fixative for at least 24 h and then the tissue was withdrawn and allowed to settle in a dehydrator (model JJ-12 J; Wuhan Junjie Electronics Co., Ltd.,) where it underwent dehydration with a gradient ethanol. These dehydrated samples were then fixed in an embedding machine (model JB-P5; Wuhan Junjie Electronics Co., Ltd.,). The embedded paraffin segments were placed on a microtome (model RM2016; Shanghai Leica Instrument Co., Ltd.,) for sectioning; each section was 3 μm thick. Finally, after dewaxing, staining, and photographing the samples' sections, the cross-sectional anatomy of leaves was measured by using NanoZoomer Digital Pathology (NDP.viewer 2.0 software).

### Physiological Measurements

Photosynthetic parameters of the second functional leaf (counting from the top downward) of plants that developed in the greenhouse were measured by Li-6400 photosynthesis measurement system (Licor Corporation, USA). The photosynthetic measurements were taken at a constant air flow rate of 500 μmol·s^−1^ and photosynthetic photon flux density of 1,000 mmol (photon) m^−2^·s^−1^. All measurements were made between 09:00 am and 11:00 am, with each repeated five times.

Relative water content of leaves was calculated according to Meher et al. (2018). Their total chlorophyll was extracted with methanol (0.1 g FW/5 ml, final dilution) and measured using the equations recommended by Wellburn (1994).

Superoxide dismutase (SOD) and malondialdehyde (MDA) were chosen as bioindicators for evaluating the extent of oxidative damage in leaf cells (Heath and Packer, 1968). Absorbance at 532 nm and 600 nm was recorded to assess the content of MDA in a given mulberry plant. The SOD activity was quantified as described in Singh et al. (2018) and detected by recording the rate of reduction of nitroblue tetrazolium (NBT).

Hormone concentrations were determined with a kit manufactured by Suzhou Keming Biotechnology Co., Ltd and assessed with the enzyme-linked immunosorbent assay (ELISA) method. The ELISA measuring precept is based on a competitive reaction and chromogenic reaction, for which a quantitative analysis was performed (Prado et al., 2014; Yang et al., 2020).

### Transcriptome Sequencing and Data Analysis

A minimum of three independent samples were randomly selected from each group for RNA-seq. Total RNA was extracted with a Plant RNA Kit (Omega Biotek, Guangzhou, China). For each sample, an mRNA library was sequenced using Illumina HiSeq2000 (150-bp paired ends); the de novo assembly was performed with Trinity (Trinity-v2.5.1), and 43, 029 unigenes were obtained. The ensuing reads per sequence tag were then mapped to the transcriptome consensus sequences in Bowtie software (Langmead et al., 2019). Annotation of gene sequences were made using Non-Redundant Protein Sequence Database (Nr), Nucleotide Sequence Database (Nt), Pfam, Cluster of Orthologous Groups for Eukaryotic Complete Genomes (KOG), Swiss-prot, Kyoto Encyclopedia of Genes and Genomes (KEGG), and Gene Ontology (GO). The number of mapped clean reads for each gene was counted and normalized using the “DESeq” package, in R software (Anders and Huber, 2010; Cho et al., 2016). Fold change *(*${\text{log}}_{2}^{\left[foldchange\right]}$ >*1)* and binomial tests were used to identify the differentially expressed genes (DEGs) in each sample. The false discovery rate *(FDR* < *0.01)* calculated *via* DESeq was applied to identify the threshold for the *P*-values in the binomial tests and analyses. Descriptions of gene functions and enriched metabolic pathways and signal transduction pathways were, respectively investigated by conducting *GO* and *KEGG*, respectively. In addition, data were further filtered by coefficient of variation (sv > 0.5). The correlation of genes was determined by the expression levels of genes and clustering trees were constructed and modules were divided following parameters (minModuleSize = 30, mergeCutHeight = 0.25). Hierarchical clustering was performed using the H-clust R package. The co-expression network construction was performed using the Weighted Gene Co-expression Network Analysis (WGCNA) R-package.

### Widely-Targeted Flavonoid Metabolomics Testing and Metabolomic Data Analysis

All leaf samples were extracted with methanol and extracts were subjected to metabolomics analysis. The method of flavonoid metabolomics extract was according to Chen et al. (2013). Metabolite identification was referenced to MassBank (http://www.massbank.jp/), KNAPSAcK (http://kanaya.naist.jp/KNApSAcK/), HMDB (http://www.hmdb.ca/)(Wishart et al., 2013), MoTo DB (http://www.ab.wur.nl/moto/), and METLIN (http://metlin.scripps.edu/index.php) (Zhu et al., 2013). Data were acquired and analyzed using Analyst 1.6.3 (AB Sciex).

### Cuticular Wax Extraction and Wax Load Measurements

Cuticular extraction was followed by Li et al. (2019). In brief, leaves were immersed twice in CHCl_3_ (50 ml) for 1 min, and then 20 microgram of n-tetracosane (C24 alkane) was added as an interior criterion, after which the wax mixtures were condensed under a flow of nitrogen and then transported to Gas Chromatography (GC) vials. To determine the wax load, we followed the methodology presented by Li et al. (2019) and Mamrutha et al. (2010b). Ultimately, the wax chemical compounds were resolved in 1000 ul of CHCl_3_ for analysis by gas chromatography-mass spectrometry (GC-MS) that was run on a GCMS-QP2010 system (Shimadzu, Japan). The amount of total wax was expressed per unit of leaf area and the latter obtained with a leaf area meter (model Yaxin-1241, Beijing Yaxin Liyi Technology., Ltd.,).

#### Quantitative Real-Time PCR (q-PCR)

All RNA was extracted from the leaves using an RNAprep Pure Plant Kit (Axygen, Beijing, China), after which the corresponding first-strand cDNA was generated by cDNA synthesis kits (TaKaRa, Beijing, China). SYBR Premix Ex Taq (TaKaRa, Beijing, China) and two-step q-PCR procedure were used for the q-PCR assays. The M. alba actin gene was used as q-PCR reference gene and the experiments were repeated three times. Selected genes and their primer sequences are listed in Supplementary Table 1.

### Data Analysis

Data analysis was performed on Microsoft Excel 2010 and variance analysis was analyzed using IBM (v21.0, IBM, USA). Adobe Illustrator CC (https://adobe-illustrator.en.softonic.com/) was used for drawing graphs. In addition, the TBtool (https://github.com/CJ-Chen/TBtools/releases) was used to draw the heatmap, and the box plot figure was performed by Origin 8.0 (Origin Software, USA) software (https://www.originlab.com/). To visualize the metabolomic data among the different samples, principal component analysis (PCA) and hierarchical cluster analysis (HCA) were implemented in R software (www.r-project.org/).

Accession numbers: The FASTQ sequence data are available from the NCBI BioProject under accession number SUB9594105.

## Results

### Physiological Changes of Mulberry Under Drought-Stress Treatment

Most mulberry cultivars (over 66%) are dioecious, and these cultivars are nearly always maintained and propagated by the grafting methods. In order to observe the real response of different ploidy roots to imposed drought stress and bypass the interaction between cultivar scion and hybrid diploid-seeding rootstock, own-rooted stem cuttings were tested in the study (Supplementary Figure 1A). The DNA content *of* the leaf cells in every tested plant were confirmed by flow cytometry. A sharp peak in nuclear DNA content in all triploids was evident, being 1.5 times that of the diploid (Supplementary Figures 1B,C).

**Figure 1:**
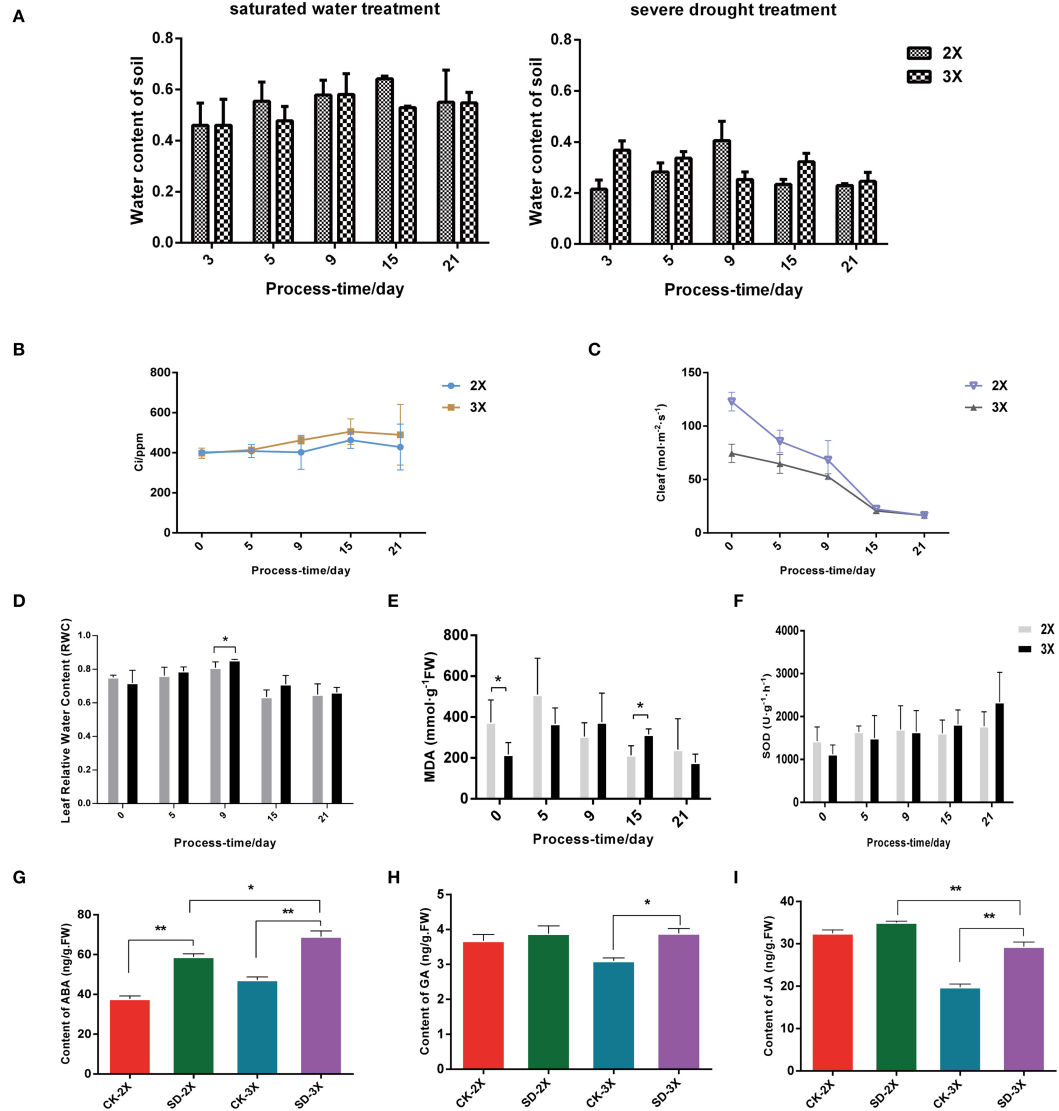
Physiological parameters in diploid (2X) and autotriploid (3X) subjected to different days of drought stress. **(A)** Water content of soil; **(B)** Ci: Intercellular CO2 concentration; **(C)** Cleaf: stomatic conductance; **(D)** Leaf relative water content; **(E)** MDA: malonaldehyde; **(F)** SOD: superoxide dismutase. **(G-I)** Hormonal content in leaves of diploid and triploid with drought stress for 9 days for ABA **(G)**, GA **(H)** and JA **(I)**. All assays were carried out at least three times and statistical significance levels were calculated using Student's *t*-test (^*^P ≤ 0.05; ^**^P ≤ 0.01). CK, control; SD, severe drought.

Water deficits can slow the growth of plants, damage their cells' morphology, and sharply lessen their moisture content, leading to plant lodging or wilting leaves. Firstly, the physiological parameters for tolerance to drought stress was measured in the diploid and triploid mulberry. This revealed higher intercellular carbon dioxide concentration (Ci) (Figure 1B) and leaf relative water content (RWC) (Figure 1D), but a lower stomatal conductance (Figure 1C) in the triploid than the diploid. It is suggested that rubisco's carboxylation may be promoted, leading to less carbon starvation and delayed drought symptoms. The MDA content increased at first, then decreased in both diploid and triploid (Figure 1E). In addition, SOD levels continued to increase under severe drought stress in the triploid, yet there was no significant change in the diploid (Figure 1F). RWC is an indicator for gauging the intensity of tolerance to drought in plants. RWC in the triploid compared with the diploid was already significantly higher after 9 days of drought treatment (Figure 1D).

Subsequently, the content of several hormones related to stress and growth in the diploid and triploid were measured. The ABA, jasmonate (JA), and gibberellin (GA) contents increased in both cultivars (Figures 1G–I) through 9 days of drought; moreover, there were significant differences in the ABA content between triploid and diploid plants after drought stress. These results suggested that ABA hormone signaling genes contribute to drought tolerance in *Morus*. Moreover, the content of zeatin riboside (ZR) also increased under the drought conditions (Supplementary Figure 2).

### Differences of Transcriptomic Data of Diploids and Triploids

**Figure 2:**
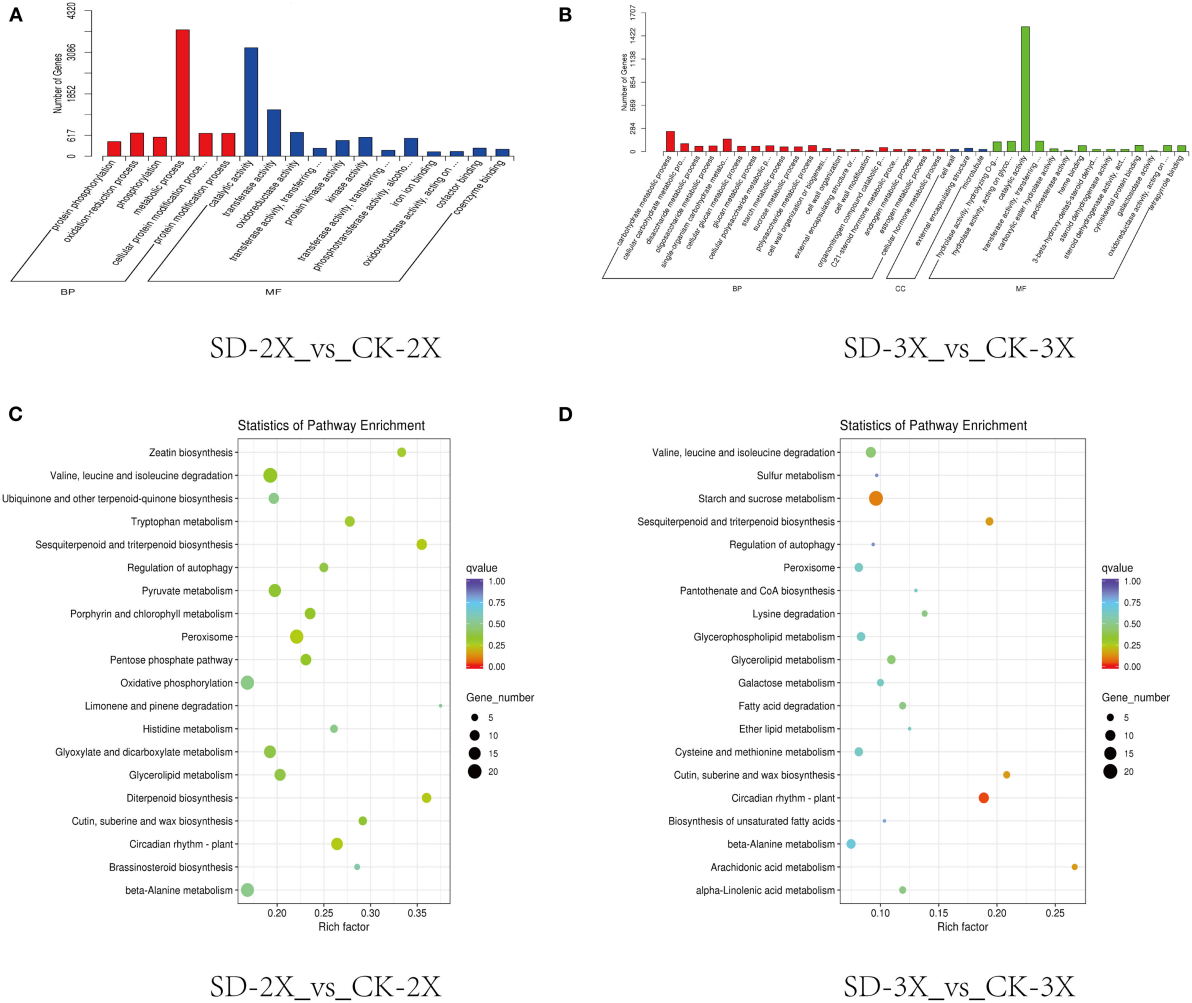
Transcriptome analysis of diploid (2X) and triploid (3X) in response to drought stress. **(A)** GO classification analysis of DEGs in diploid (2X) response to drought stress. **(B)** GO classification analysis of DEGs in triploid (3X) in response to drought stress. **(C)** The top 20 enriched pathway in drought stress conditions in diploid (2X). **(D)** The top 20 enriched pathway in drought stress conditions in triploid (3X). The size and the color of solid circles represent the number of transcripts involved in certain pathways and the significant value (q-value) of the rich factor, respectively. CK, control; SD, severe drought.

The samples for transcriptome sequencing were taken at four time points (0, 5, 9 days, and 15 days) (Supplementary Table 2), with three replicates, to construct the cDNA libraries. The overview of the RNA-Seq result is shown in Supplementary Table 2. To decipher the potential processes distinguishing triploid and diploid responses to drought stress, the up-regulated DEGs at each of the three drought stress stages were analyzed by GO and KEGG enrichment analyses. Notably, the DEGs on day 15 showed that “metabolic process” and “catalytic activity” were the two highest enriched GO items in both the diploid and triploid (Figures 2A,B). From the Sever Drought-2X_vs_Control Check-2X (SD-2X_vs_CK-2X) pairwise comparison, the two most enriched pathways were peroxisome and oxidative phosphorylation (Figure 2C). However, the starch and sucrose metabolism pathway was enriched in the triploid under drought stress vs. control conditions (Figure 2D). From the overall trend in Figure 1, the content of intercellular CO_2_ increased in both the diploid and triploid; moreover, the triploid had lower stomatal conductance but higher intercellular CO_2_ as compared with the diploid. Carbon dioxide fixation accounts for substantial primary productivity on plants. Intercellular CO_2_ may have a relationship with primary metabolic pathways, such as starch and sucrose metabolism. Sucrose-phosphate synthase (E2.4.1.14) was up-regulated and endoglucanase (E3.2.1.4) was downregulated, in both the diploid and triploid. The 1,4-alpha-glucan branching enzyme (E2.4.1.18) was upregulated in the triploid while starch synthase (EC:2.4.1.21) was upregulated in the diploid. Additionally, fructokinase (E2.7.1.4) was downregulated in the triploid whereas both hexokinase (EC:2.7.1.1) and alpha-amylase (EC:3.2.1.1) were downregulated in the diploid (Supplementary Figure 3).

**Figure 3:**
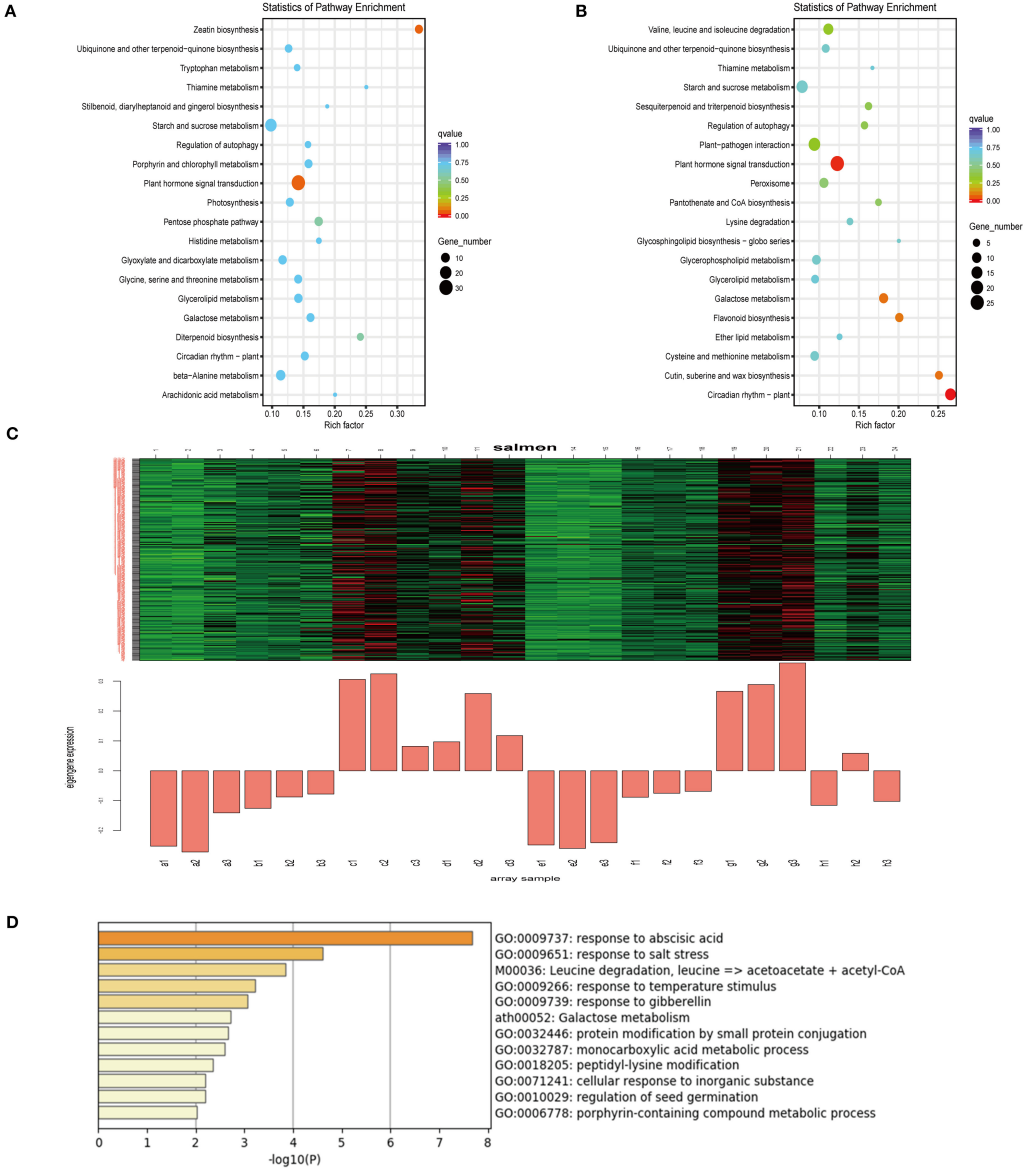
KEGG enrichment analysis of the DEGs on the 9th day and Weighted gene co-expression network analysis (WGCNA) of differentially expressed genes (DEGs) identified in drought treated and control over three sampling time points (5, 9, and 15 days) during drought stress. **(A,B)** The 20 most significantly enriched KEGG pathways for DEGs in response to drought after nine days in diploids (2X) **(A)** and triploids (3X) **(B)**. **(C)** Salmon module eigengene expression values across all the samples. The upper portions of **(D)** figure is heat maps of the expression values of each gene (row) in each sample (column): red—highly expressed; green—lowly expressed; black—neutral; Control: (a: CK-2X, e: CK-3X); Severe drought with 5 days: (b:SD-2X-5, f:SD-3X-5); Severe drought with 9 days: (c: SD-2X-9, g: SD-3X-9); Severe drought with 15 days: (d: SD-2X-15, h: SD-3X-15). CK: control; SD: severe drought. **(D)** Pathway and process enrichment analysis. Each term is represented by a circle node, where its size is proportional to the number of input genes that fall into that term, and its color represent its cluster identity.

To further elucidate the transcriptome response of mulberry trees to drought, RNA-Seq data were reanalyzed using weighted gene co-expression network analysis (WGCNA). The upregulated DEGs with 9 days of drought were analyzed by KEGG, which revealed plant hormone signal transduction pathways that were enriched (Figures 3A,B). The top 5,000 expressed genes sorted by FPKM were selected. According to the correlations between the modules and samples, we found that the diploid and triploid samples were correlated to some extent with the salmon module: generally, genes of this module were highly expressed in the samples (c: SD-2X-9, d: SD-2X-15, g: SD-3X-9, h: SD-3X-15) in the late-stage of drought (Figure 3C, Supplementary Table 2). The cytoHubba plugin of Cytoscape was used to analyze the hub genes in the PPI network, and the following genes with the top ten grades were identified as hub genes: XM_010092529, XM_010114095 (LOC21394087), XM_010095273 (LOC21385040), XM_010105275 (LOC21405851), XM_010091874, XM_010099774 (LOC21408873), XM_010115192 (LOC21409471), XM_010109610, XM_010110196 (LOC21393980), and XM_010109141 (LOC21398866) (Supplementary Table 5). We randomly selected XM_010109141 (LOC21398866) to perform quantitative RT-PCR (q-PCR) (Supplementary Figure 3B). We further annotated the gene functions within the salmon module, yielding 159 annotated unigenes whose uniprot IDs were input into the Metascape online analysis software (https://metascape.org/gp/index.html#/main/step1). Using *Arabidopsis* as the reference species, 108 of those genes were annotated and enriched. Genes in this module were most enriched in terms of the ABA response and salt stress response (Figure 3D). Next, seven DEGs (LOC21408186, NCED3, LOC21405962, LOC21407792, LOC21404511, LOC21412472, and LOC21397445) which associated with the signaling network of ABA, JA, and GA were compared for their expression levels in triploids and diploids under drought conditions vs. the control. All these genes were up-regulated in both the triploid and diploid (Supplementary Figure 4). One of those genes, *Morus* probable carotenoid cleavage dioxygenase 4 (*LOC21405962*), is related to the synthesis of abscisic acid aldehyde, which exhibited extremely significant difference in the triploid compared with the control (Supplementary Figure 4E), implying that ABA hormonal signaling might be an important factor affecting stress defense and polyploid morphology after increasing the number of chromosomes. Moreover, two crucial genes were significantly upregulated that encoded TSPO (an outer membrane tryptophan-rich sensory protein) and the CAP160 protein (LOC21398866), which are involved in the drought response of plants and enable activation of the ABA response and salt stress response pathway. Furthermore, the ABA response gene ABRE-*bind factor* (*ABF*) (Supplementary Figure 3C) also showed a positive interaction with drought but the *9-cis-epoxycarotenoid dioxygenase* (*NCED*) (Supplementary Figure 4D) was down-regulated by drought. However, the mechanisms responsible for the effects of ABA regulation on plant growth and defense stress still need to be studied.

### Impact of Drought Stress on Flavonoid Biosynthesis in Mulberry

**Figure 4:**
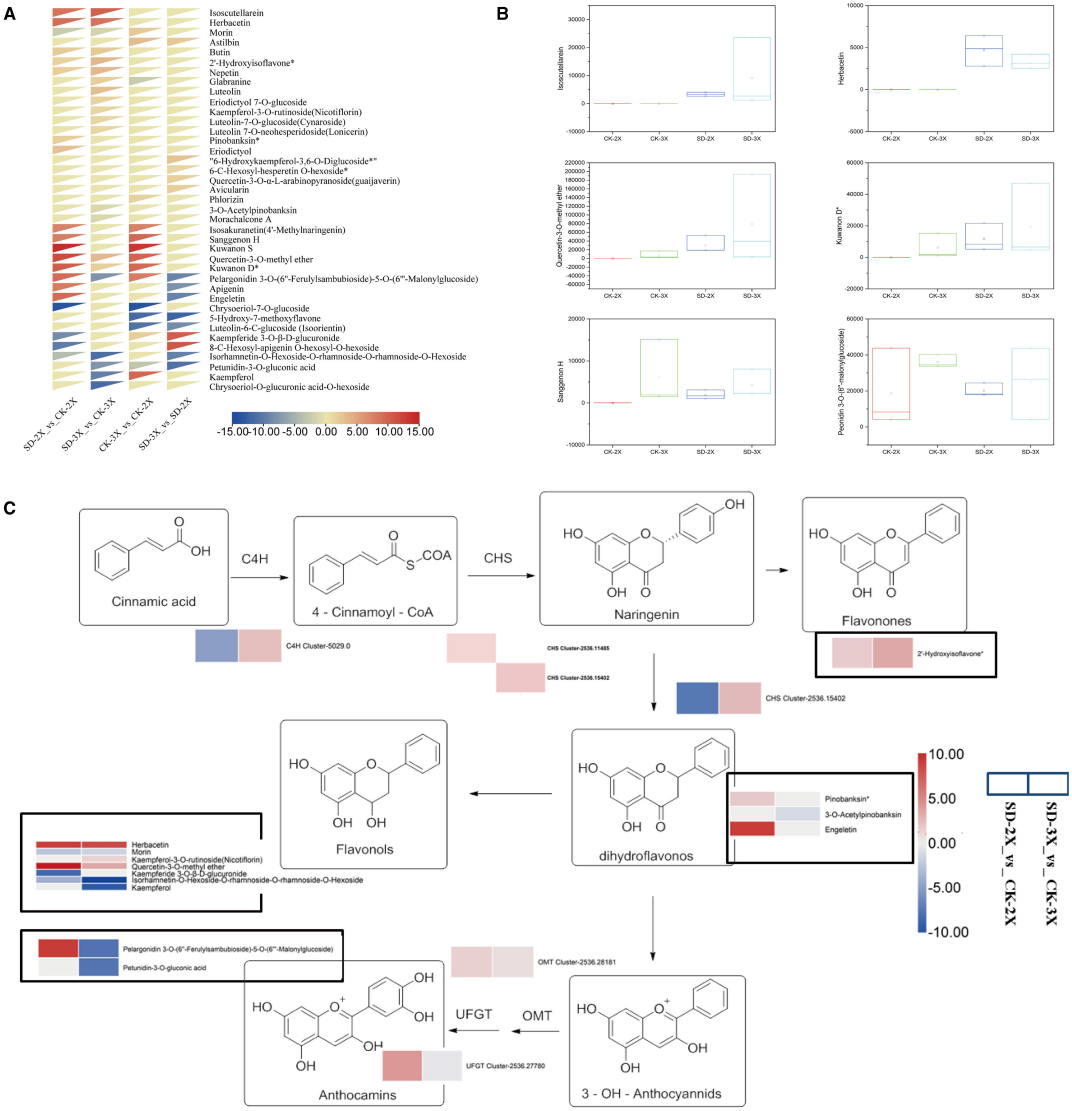
Differentially accumulated metabolites identified from diploid (2X) and triploid (3X) involved in the flavonoid biosynthesis under drought stress and overview of the major metabolic and transcript changes response to drought stress. **(A)** Heatmap hierarchical clustering showing metabolite fold change in diploid (2X) and triploid (3X) in comparison with the control. The color scale for hierarchical clustering is labeled. The scale bar displays fold change values. **(B)** Box plot for the temporal variability of key flavonoid compound signal intensities in all samples. **(C)** Overview of the major metabolic and transcript changes response to drought stress in diploid (2X) and triploid (3X). Arrows show the metabolic stream. The genes and metabolites that are up-regulated and down-regulated are shown in red and blue, respectively. Circled in black is the metabolite data. The scale bar displays fold change values. Abbreviations: cinnamic acid-4-hydroxylase, C4H, chalcone synthase; CHS, UDP-glucose favonoid-3-O-glucosyltransferase; UFGT, O-methyltransferase; OMT. CK, control; SD, severe drought.

We found that the metabolism process was enriched in DEGs of mulberry in response to drought (Figure 2). Changes of flavonoid metabolites which possess properties of oxidation-reduction in their leaves at 15 days of drought were analyzed. Overall, 124 metabolites' intensities were obtained *via* the chosen reaction monitoring (SRM) of the liquid chromatography-tandem quadrupole mass spectrometry (LC-QqQ-MS) analysis (Supplementary Table 3). Next, all 124 metabolites were subjected to principal component analysis (PCA) to gain further insight into the contribution of various metabolites in the diploid and triploid response to the drought (Supplementary Figure 5). All 124 metabolites were loaded onto the two major principal components (PC1 and PC2), which explained 60.63% of the variance in the data (Supplementary Figure 5). To obtain a better metabolite segregation, a log2 transformation of metabolite fold-changes was performed, and the resulting information matrix was analyzed using heatmap hierarchical clustering (Figure 4A). A clear segregation between drought effects and species was evinced by the heatmap. Several metabolites were induced in the diploid genotype, especially isosakuranetin (4'-methylnaringenin), sanggenon H, kuwanon S, apigenin, and engeletin (Figure 4A). However, six flavonoid compounds, glabranine, luteolin, eriodictyol 7-O-glucoside, kaempferol-3-O-rutinoside (nicotiflorin), Luteolin-7-O-glucoside (Cynaroside), and luteolin 7-O-neohesperidoside (lonicerin), were exclusively detected in the triploid when compared with its control (well-watered). Among all differential metabolites found, isoscutellarein and herbacetin were the most highly accumulated ones in both genotypes (Supplementary Table 4). In comparison with their well-watered counterparts, isorhamnetin-O-hexoside-O-rhamnoside-O-rhamnoside-O-hexoside was decreased in both the diploid and triploid mulberry plants (Figure 4).

To identify the genes influencing the high accumulation of flavonoids in the diploid and triploid while *under* drought stress, those flavonoid-related genes in the transcriptome results were mapped to the flavonoid pathway of KEGG (http://www.genome.jp/kegg/). The DEGs and differently accumulated compounds were positioned on the flavonoid synthesis pathway. RNA-Seq analysis revealed that the log2 (fold change) value of the chalcone synthase (*CHS*) was increased by drought stress for both the diploid and triploid. The expression of the *CHS* gene was verified by RT-PCR, using the β-actin gene as an internal reference. In the diploid, *CHS* was upregulated 97.52-fold, while it increased just 1.06-fold in the triploid (Supplementary Figure 6). The differential fold-change in gene expression based on RT-PCR deviated slightly from obtained by RNA-Seq, but the patterns in gene expression revealed by those two methods was the same. Isoscutellarein and herbacetin, in the diploid and triploid, show similar trends in response to drought, but vice versa for the trend in pelargonidin (Figure 4B). The levels of some reducing compounds, such as dihydroflavonols and anthocyanins, were not elevated in triploid, matching the RNA-Seq results (Figure 4C). In summary, we demonstrated that flavonoid metabolites' variance in a diploid and triploid was responsive to drought; hence, our results point to the importance of the *CHS* gene in the formation and progression of flavonoids in diploid mulberry.

### Effects of Drought Stress on Cuticular Wax Load and Surface Barriers of Mulberry Leaves

**Figure 5:**
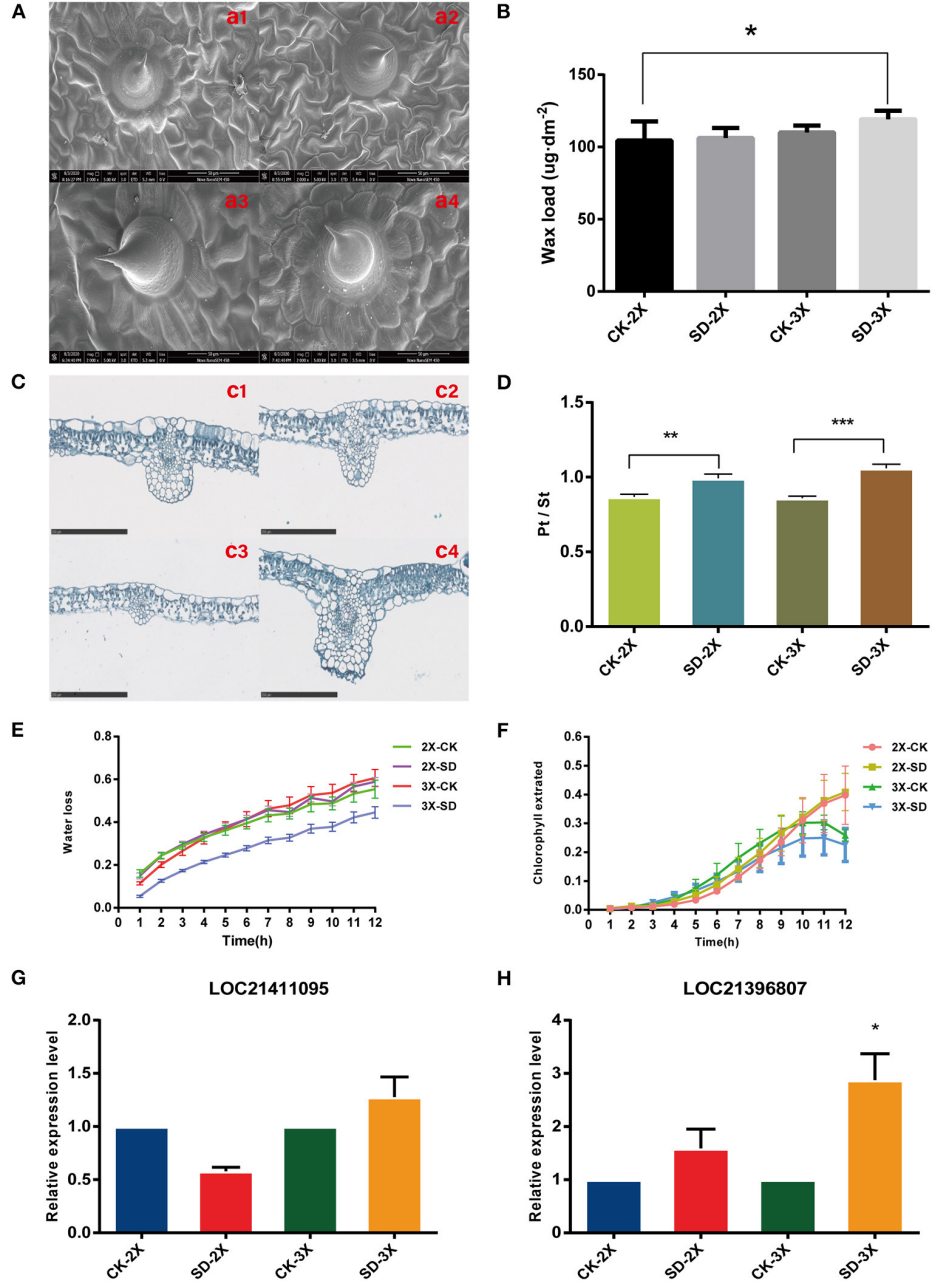
Surface wax load, surface permeability, and histological traits of leaf in diploid (2X) and triploid (3X). **(A)** Morphological display of surface wax in the pressure side of leaf of diploid (2X) and triploid (3X) under two water treatments observed using scanning electron microscopy (SEM). Ample water treatment: (a1) CK-2X; (a3) CK-3X; Severe drought stress: (a2) SD-2X; (a4) SD-3X. **(B)** The wax coverage changes of leaf during water deprivation treatment for 9 days. **(C)** Histological characterization of leaves of diploid (2X) and triploid (3X) in well-watered and drought stress for 9 days. Ample water treatment: (c1) CK-2X; (c3) CK-3X; Severe drought stress: (c2) SD-2X; (c4) SD-3X. Bar = 250 um. **(D)** Palisade tissue-spongy tissue ratio of two cultivars. Surface permeability of leaves with drought stress. **(E)** Water loss rates and **(F)** chlorophyll extraction yields. Values represent means of three replicates. Error bars = SD. **(G,H)** Quantitative real-time PCR analysis of LOC21411095, LOC21396807 expression between diploid (2X) and triploid (3X) with drought stress, which related to wax synthesis. All assays were carried out at least three times and statistical significance levels were calculated using Student's *t*-test (^*^P ≤ 0.05; ^**^p ≤ 0.01; ^***^p ≤ 0.001). CK, control; SD, severe drought.

The Figure 1 results indicated that the RWC of diploid and triploid leaves was significantly different for day 9 of the drought treatment, and wax synthesis pathway in triploid was enriched and continuously so in day 9 and 15 (Figures 2D, 3B). Accordingly, further investigations were conducted on two cultivars under drought stress for 9 days. The stomata closure was induced in mulberry leaves (Supplementary Figure 7) and their palisade tissue-spongy tissue ratio increased in both cultivars under drought stress (Figures 5C,D). The palisade tissue-spongy tissue ratio increasing may be one reason why triploids can withstand drought stress. Using SEM (Scanning Electron Microscopy), we found the surface of mulberry leaves covered with membranous wax (Figure 5A). Moreover, an increase in this wax content in the epidermis was induced by drought (Figure 5B), with more wax content on the triploid than diploid leaves. This finding implied that a large amount of waxiness of the epidermis is one way that triploids prevent water loss. Further assays showed that the water loss rate (45%) in the triploid was lower than that of its diploid progenitors (59%) After 12 h, the triploid leached chlorophyll less rapidly from intact leaves only up to 25%, vs. diploid up to 32% for the after 10 hr (Figures 5E,F). However, the water loss rates and leached chlorophyll rates changed little between the drought treated and well-watered (control) diploid plants. Furthermore, two genes associated with long-chain fatty acid (LCFA) were tested, which were up-regulated *under* drought in triploid (Figures 5G,H). Taken together, these results demonstrated that, with an enlarged genome, there is a resultant increase in cuticular wax production and deposition, capable of enhancing the leaf surface barrier properties of mulberry.

### Autophagy Pathways and Transcription Factors Participate in Mulberry Drought Response

**Figure 6:**
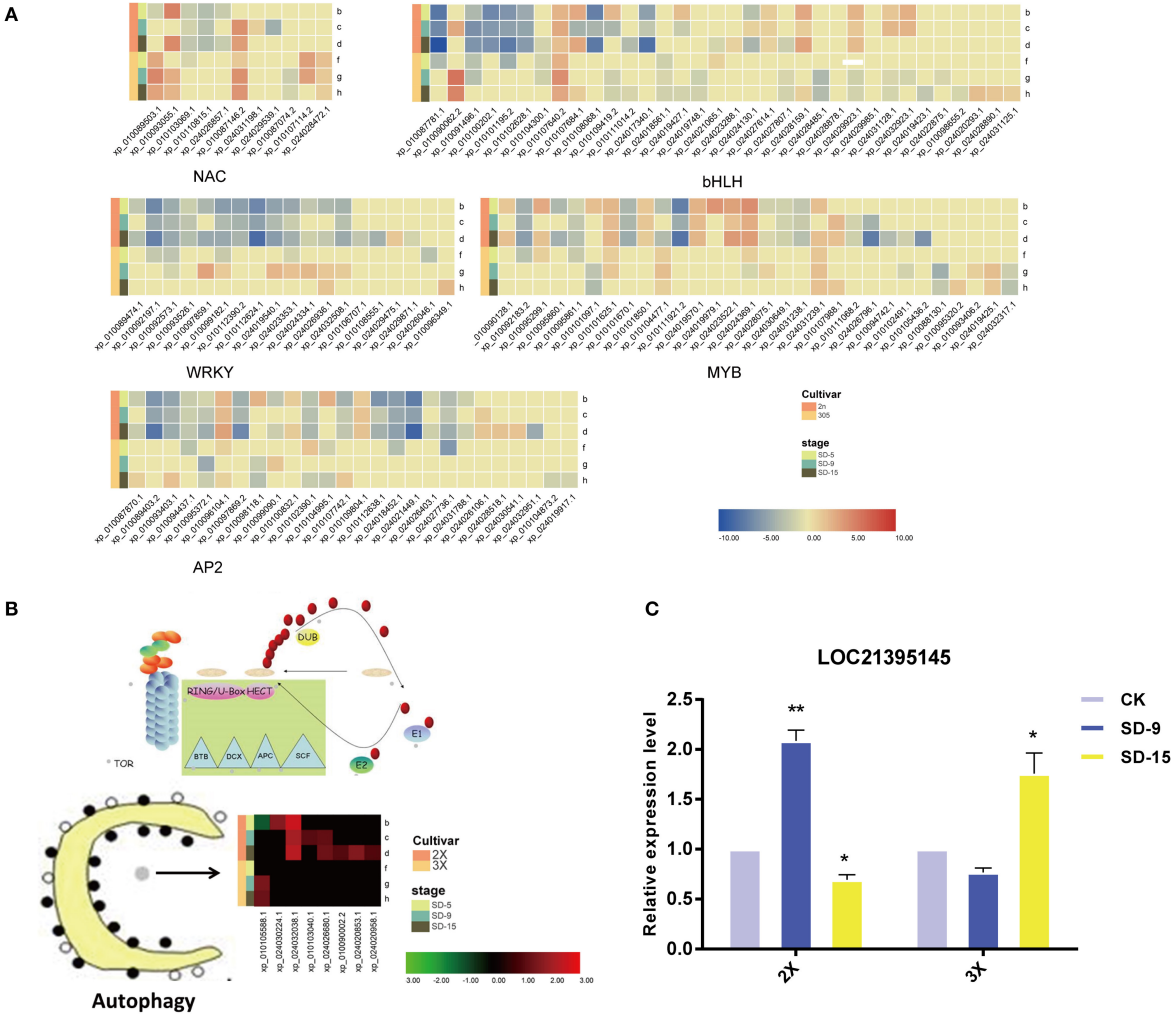
Heatmap of transcription factor and autophagy related transcripts. **(A)** Heatmap of gene expression for TFs enriched. **(B)** Heatmap of gene expression for eight DEGs enriched in the ‘autophagy’ pathway. Heatmap color indicates fold change of expression under drought compared with that in well-watered of both diploid (2X) and triploid (3X). **(C)** Quantitative real-time PCR analysis of *LOC21395145* gene of diploid (2X) and triploid (3X) identified in the drought treatment and control over two sampling time points (9 and 15 days). All assays were carried out at least three times and statistical significance levels were calculated using Student's *t*-test (^*^,*p* ≤ 0.05;^**^,*p* ≤ 0.01). CK, control; SD, severe drought.

The autophagy pathway was always among the top 20 pathways most significantly enriched for DEGs in all comparisons-according to the KEGG analysis, thus implying that autophagy could be relevant for the drought response of the mulberry diploid and triploid trees. More specifically, the number of autophagy-related genes responsive to drought in the diploid exceeds that in triploid. We found that, under the drought treatment, a gene encoding (*ATG*) was upregulated to higher levels by day 9 and 15 in the diploid and triploid, respectively (Figures 6B,C).

In this study, many TF genes were prominently induced by drought stress, and they showed different expression levels between diploid and triploid. The significantly differentially expressed TFs were *MYB, bHLH, WRKY, AP2*, and *NAC* TFs, which are associated with tolerance and stress stimulation (Figure 6A). More TFs participated in the diploid's response to drought than in the triploid. One *MYB* gene (XM_010102795.2/XP_010101097.1) was randomly selected for RT-PCR (Supplementary Figure 5C). This showed it was downregulated by drought in the triploid, but with upregulated expression in the diploid, which is broadly in line with the RNA-seq results.

## Discussion

### Enhanced Drought Tolerance in the Triploid

**Figure 7:**
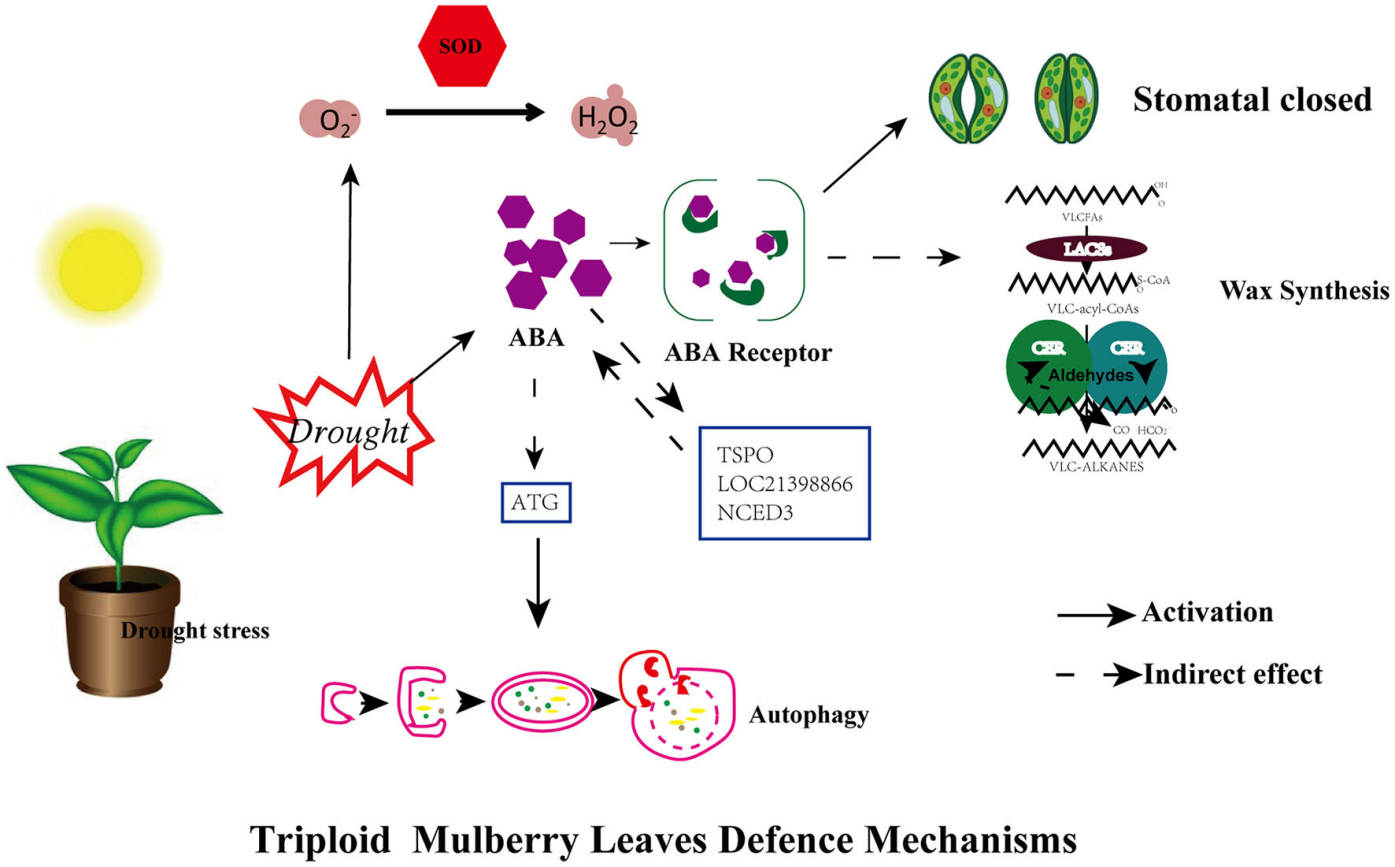
A model for mechanisms underlying the enhanced drought tolerance of mulberry triploid (3X). Under drought stress, triploid plants exhibit enhanced drought tolerance in comparison with diploid progenitors due to the activation of multifaceted defense machinery in leaves. For the first, activation of ABA hormonal signaling and antioxidant enzyme (SOD) leads to efficient hormonal signaling and improved ROS scavenging ability, and ABA-induced stomatal closure concurrently. Secondly, some genes (TSPO, NCED3, and LOC21398866) and ATG gene related to ABA signaling showed significant upregulation in expression. Autophagy is regulated by autophagy-related (ATG)gene, meanwhile, wax load increased.

Own-rooted stem cuttings ShinIchinose (2X) and Shaansang 305 (3X) were used in this research, of which the triploid is a valuable autotriploid mulberry tree that is currently the *better* cultivar for production and application purposes. Based on the above analysis and results, we could propose a potential mechanism to explain the enhanced drought tolerance in the triploid compared to the diploid (Figure 7). Since the available genome for mulberry was yet to be assembled when we did our analysis, we chose non-mulberry transcriptome analysis (Jiao et al., 2020). Although we carried out the experiments using saplings, the discoveries may be directly applicable to a broader range of development stages and stress scenarios in this species. Knowledge derived from further studies of the genes uncovered in this study may also prove valuable to breeding, possibly enabling ways to devise new strategies for mulberry to respond to the twin challenges of ongoing drought and groundwater depletion in changing environments.

The results showed that survivability of diploid and triploid under drought stress is associated with cytotypes in this study. In the triploid, ABA signaling was substantially altered in response to ABA accumulation. Furthermore, the signals associated with the ABA response showed significant changes, such as stomatal closure, upregulation of autophagy-related gene (*ATG*), and more of a wax load under water-restricted growing conditions. The expression of several genes (*TSPO, NCED3, and LOC21398866*) related to ABA signaling showed significant upregulation. Moreover, *TSPO* is associated with not only the ABA signaling pathway but also autophagy degradation (Guillaumot et al., 2009; Vanhee and Batoko, 2011; Vanhee et al., 2011; Hachez et al., 2014), hence this gene deserves in-depth study. Nonetheless, gene transformation in mulberry is currently not possible because woody plants' particularities and technical limitations.

Promising research topics, or “hotspots,” are put forward after comparing complex issues through standardized analysis and comprehensive judgment of drought resistance in polyploid mulberry trees. Firstly, our results are in line with some previous reports which indicated polyploid plants have higher drought tolerance than diploids (Wei et al., 2019; Xiao et al., 2019b), which suggests that divergence in drought-tolerance traits between cytotypes is a probable adaptive mechanism for polyploidy survival in arid regions, although explanations for other species' resistance cannot be ruled out. Secondly, Shinichinose (2X) increase the functional metabolites (some reducing flavonoids such as dihydroflavonos and anthocamins) content when exposed to drought stress. The flavonoid metabolism involved in drought resistance identified here could be of great value to medicinal plants and may have valuable insights for other species as well. Thirdly, our findings strengthen the need to identify which adaptations might facilitate the transition from diploidy to polyploidy. Overall, it should allow the integration of the diploidy-polyploidy cycle into an evolutionary model for eukaryotes (Comai, 2005).

### Parallel Response to Drought Stress Between Diploid and Triploid Mulberry

Our results showed that several traits related to drought tolerance exhibited a similar tendency in both cultivars. Our results did show that the triploid has a higher content of ABA as well as JA and GA compared with diploid under drought stress. It is consistent with the reactions in most plants in response to drought (Chen et al., 2019). Plants perceive the intracellular signaling induced by drought, which elicits the production of ROS and NO and stimulates key plant hormones, such as ABA, SA, and JA, leading to accumulated osmoregulatory compounds and stomatal closure (Agurla et al., 2018). In response to drought stresses, the role of ABA is generally very pronounced: a sharp rise in plant ABA levels and corresponding activation of ABA-responsive signaling consequence result in the regulation of stomatal aperture and the expression of stress-responsive genes (Asselbergh et al., 2008). The expression levels of ABF, MYB, and NCED genes had higher expression in the drought treatment than the control, in both mulberry cultivars. Stomatal closure, increased hormone levels, and modulated genes are closely connected. As a result, stomatal closure by ABA surged to reduce water losses and less pathogen entry throughout drought treatment (Lim et al., 2015). We found the plant–pathogen interaction pathway enriched in downregulated genes coincidentally and it existed in both diploid and triploid. Increased ABA, however, can result in suppression of the SA signaling pathway in the mesophyll cells inside the leaf, thus weakening post-invasion, SA-mediated resistance (Jiang et al., 2010). A number of reports, however, support a negative function of ABA in plant immunity (Cao et al., 2011). Decreased resistance to pathogens in plant may be driven by increased ABA levels or ABA sensitivity, and *vice versa*.

Besides, mounting reports that characterize interactive and hostile interactions between abiotic and biotic stress reactions point to ABA as an indispensable component in integrating and tweaking abiotic and biotic stress-response signaling networks (Asselbergh et al., 2008). Experiments that investigate the relationship between the ABA independent pathway will be performed in the future.

### Differences in Drought Tolerance Between Triploids and Their Diploid Ancestors

Divergency in drought tolerance between triploids and their diploid ancestors may advance habitat differentiation, and accordingly, the spatial separation of cytotypes. Our results are consistent with the finding that triploid or tetraploid pine showed higher SOD levels than diploid trees, on account that the SOD gene lives on chromosome and inhibits the formation of lipid peroxide (Niwa and Sasaki, 2003). In that work, it was suggested those trees could protect themselves because SOD can scavenge for oxygen radicals induced by drought stress; hence, polyploidy species should be planted (Niwa and Sasaki, 2003). Beyond that, two major discoveries were made in the present study. Foremost, we found that more cuticular wax load in the triploid than in the diploid of mulberry. Leaf cuticular wax amount and crystal morphology have previously been shown to be important for mitigating post-harvest water losses from leaves (Mamrutha et al., 2010b), but this trait has rarely been linked to variation in ploidy. Our findings agree with some previous research reporting that wax accumulation and moisture loss prevention occur in tandem, or “go together,” in that wax accumulation *had* improved plants' drought resistance (Cameron et al., 2006; Zhong et al., 2020). From data available to date, most correlation-based research on wax synthesis and resistance of polyploidy have mostly focused on wheat, with scarcely any studies on the association in woody plants. The most noteworthy change in the wax profile of drought-treated leaves is the dramatic increase of its alkane constituents; therefore, plants may increase the total leaf wax amount by synthesizing a large amount of alkane to cope with a drought event (Kosma et al., 2009). Evaluating the wax composition of the diploid and triploid of mulberry will merit more attention in follow-up studies. Secondly, we found some flavonoid compounds, such as anthocyanins and flavonols induced by drought, which were up-regulated in diploid, yet down-regulated in triploid. It is known that drought stress causes the stomata to close (Cardoso et al., 2020; Kapoor et al., 2020), the absorption of carbon dioxide decreases, meanwhile the consumption of NADPH^+^H^+^ decreases. The excess NADPH^+^H^+^ (Liu et al., 2011) makes the diploid passively produce excessive reducing substances in plant body without changing the enzyme activity (Kleinwachter and Selmar, 2015). *Presumably*, transporters ferry these resulting reductive flavones to cytoplasm and relieve peroxidation damage in cells. The ROS are cleared in the triploid by some enzymes such as SOD, but the secondary metabolic pathway does not actively participate in the clearance mechanism. The correlation between genotype and stress responsiveness varies for plant species as well. We speculate that the different performance in two mulberry genotypes may be associated with a distinct drought susceptibility threshold in the diploid vis-à-vis the triploid (Liang et al., 2020). Precisely, obtaining reductive secondary metabolites could exert a certain degree of stress on different cultivars of mulberry trees.

Zhang et al. (2020) made a point that there is reciprocal regulation between stress-response and growth-control pathways in plants. According to a recent report, young seedlings of Arabidopsis adapt to stress by retaining starch and delaying growth *via* ABA-dependent and -independent pathways (Liu et al., 2019a). However, it is worth mentioning that, in our results, sugar-starch metabolism occurs *in the both the* up-regulated and down-regulated gene enrichment pathway at day 15 in the drought response. Sucrose-phosphate synthase (E2.4.1.14) was always up-regulated in the triploid, whose genes' transcription are likely regulated by osmotic stress and low temperature (Solis-Guzman et al., 2017; Bilska-Kos et al., 2020). Conversely, hexokinase (EC: 2.7.1.1) was always downregulated in the diploid, showing that the *Arabidopsis* hexokinase1 (*AtHXK1*) in tobacco guard cells can augment water-use efficiency (WUE) and confer tolerance to salt and drought stresses (Lugassi et al., 2019), so our results suggest diminished WUE in the diploid. Starch is a determinant of plant physical fitness in the face of abiotic stress (Thalmann and Santelia, 2017). Plants remobilize their starch reserves to release sugars, deriving metabolites and energy to help to mitigate the incurred stress. This is an indispensable process for plant productivity under challenging environmental conditions (Thalmann and Santelia, 2017).

To sum up, our results suggest the possibility that ploidy levels are associated with drought resistance, and further, that triploids may better cope with drought events. The studied mulberry diploid (ShinIchinose) may have medicinal importance after drought treatment. Although the diploid and triploid feature similar aspects in terms of their response to drought, they have different values to tree management.

## Data Availability Statement

The datasets presented in this study can be found in online repositories. The names of the repository/repositories and accession number(s) can be found in the article/Supplementary Material.

## Author Contributions

FJ conceived the Project and HL designed the experiments and managed components of the project. HL, FJ, and HS organized the manuscript. FJ, LB, RZ, MZ, CS, and YQ oversaw and participated in all the related analyses. HL, SH, and TH performed some of the sampling and experiments. All authors contributed to the article and approved the submitted version.

## Funding

The work was supported by the National Key Research and Development Project of China, China (no. 2019YFD1000600), Funds of Modern Agricultural Industrial Technology System (no. CARS-18), and the Shannxi Provincial Education Department Research Project (No. 17JS002).

## Conflict of Interest

The authors declare that the research was conducted in the absence of any commercial or financial relationships that could be construed as a potential conflict of interest.

## Publisher's Note

All claims expressed in this article are solely those of the authors and do not necessarily represent those of their affiliated organizations, or those of the publisher, the editors and the reviewers. Any product that may be evaluated in this article, or claim that may be made by its manufacturer, is not guaranteed or endorsed by the publisher.

## Supplementary Material

Supplementary Figure 1 Phenotype and flow histogram of plants **(A)** Normal growing and drought stressed of Shinichinose (2X), Shaansang-305 (3X) cultivated for 15 days. Flow histogram of **(B)** Shinichinose (2X) and autotriploid species of **(C)** Shaansang-305 (3X).

Supplementary Figure 2 Hormonal content in leaves of diploid (2X) and triploid (3X) with 9 days of drought stress for ZR **(A)**, BR **(B)**, IPA **(C)**, and IAA **(D)**. All assays were carried out at least three times and statistical significance levels were calculated using Student's *t*-test (^*^*P* ≤ 0.05; ^**^*P* ≤ 0.01). CK, control; SD, severe drought.

Supplementary Figure 3 Pathways of starch and sucrose metabolism of diploid (2X) and triploid (3X) in response to drought stress. CK, control; SD, severe drought.

Supplementary Figure 4 Quantitative real-time PCR analysis of genes expression between diploid (2X) and triploid (3X) with drought stress. All assays were carried out at least three times and statistical significance levels were calculated using Student's *t*-test (^*^*P* ≤ 0.05; ^**^*p* ≤ 0.01; ^***^*p* ≤ 0.001). CK, control; SD, severe drought.

Supplementary Figure 5 Principal component analysis (PCA) of the normalized metabolite intensities in diploid (2X) and triploid (3X). **(A)** PCA loading plot of PC1 against PC2. Each metabolite is represented by a single dot. **(B)** PCA plot showing separation by genotype. The accumulation of variance percentage is indicated in each PC. **(C)** PCA biplot of metabolite and genotype segregation.

Supplementary Figure 6 Quantitative real-time PCR analysis of LOC21411095 and LOC21396807 expression between diploid (2X) and triploid (3X) with drought stress, which related to flavonoid synthesis. All assays were carried out at least three times and statistical significance levels were calculated using Student's *t*-test (^*^*P* ≤ 0.05; ^**^*p* ≤ 0.01; ^***^*p* ≤ 0.001). CK, control; SD, severe drought.

Supplementary Figure 7 Scanning electronic microscopic observation of stomata in diploid(2X) and triploid (3X) leaves.

Supplementary Table 1 List of primer sequences used in this study.

Supplementary Table 2 Summary of RNA sequencing and alignment data for each sample.

Supplementary Table 3 Differentially accumulated metabolites identified from diploid (2X) and triploid (3X) under drought stress.

Supplementary Table 4 Heatmap hierarchical clustering showing metabolite fold change in diploid (2X) and triploid (3X) in comparison with the control.

Supplementary Table 5 The top 10 hub genes in salmon module.
