# Supplementary figures and images for: Secondary Metabolism and Hormone Response Reveal the Molecular Mechanism of Triploid Mulberry (Morus Alba L.) Trees Against Drought
Source: Front Plant Sci. 2021 Oct 6;12:720452. doi: 10.3389/fpls.2021.720452 (PMC8528201; doi:10.3389/fpls.2021.720452)

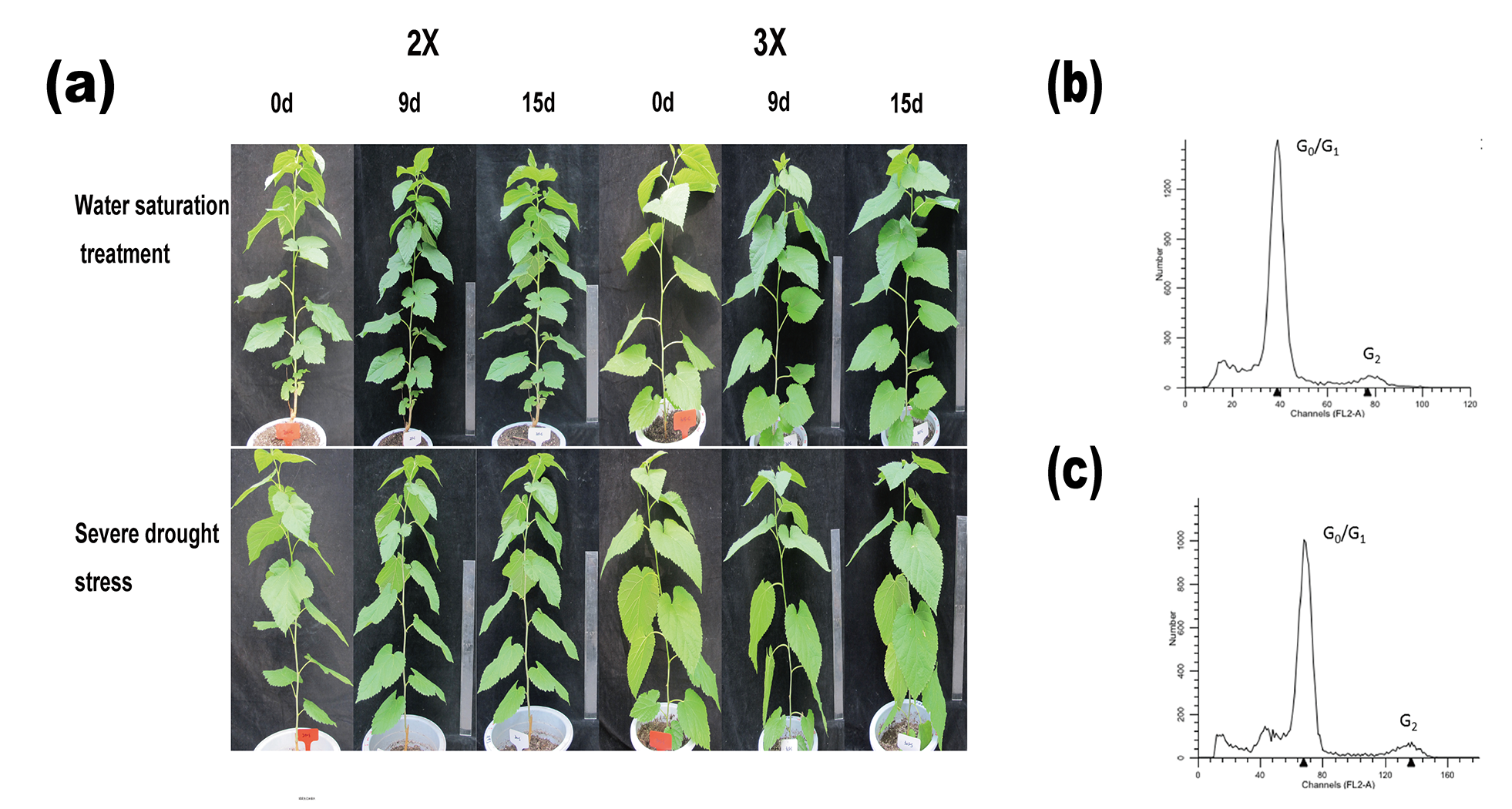

Supplement: Supplementary Figure 1 — Phenotype and flow histogram of plants (A) Normal growing and drought stressed of Shinichinose (2X), Shaansang-305 (3X) cultivated for 15 days. Flow histogram of (B) Shinichinose (2X) and autotriploid species of (C) Shaansang-305 (3X). [file Image_1.TIF]

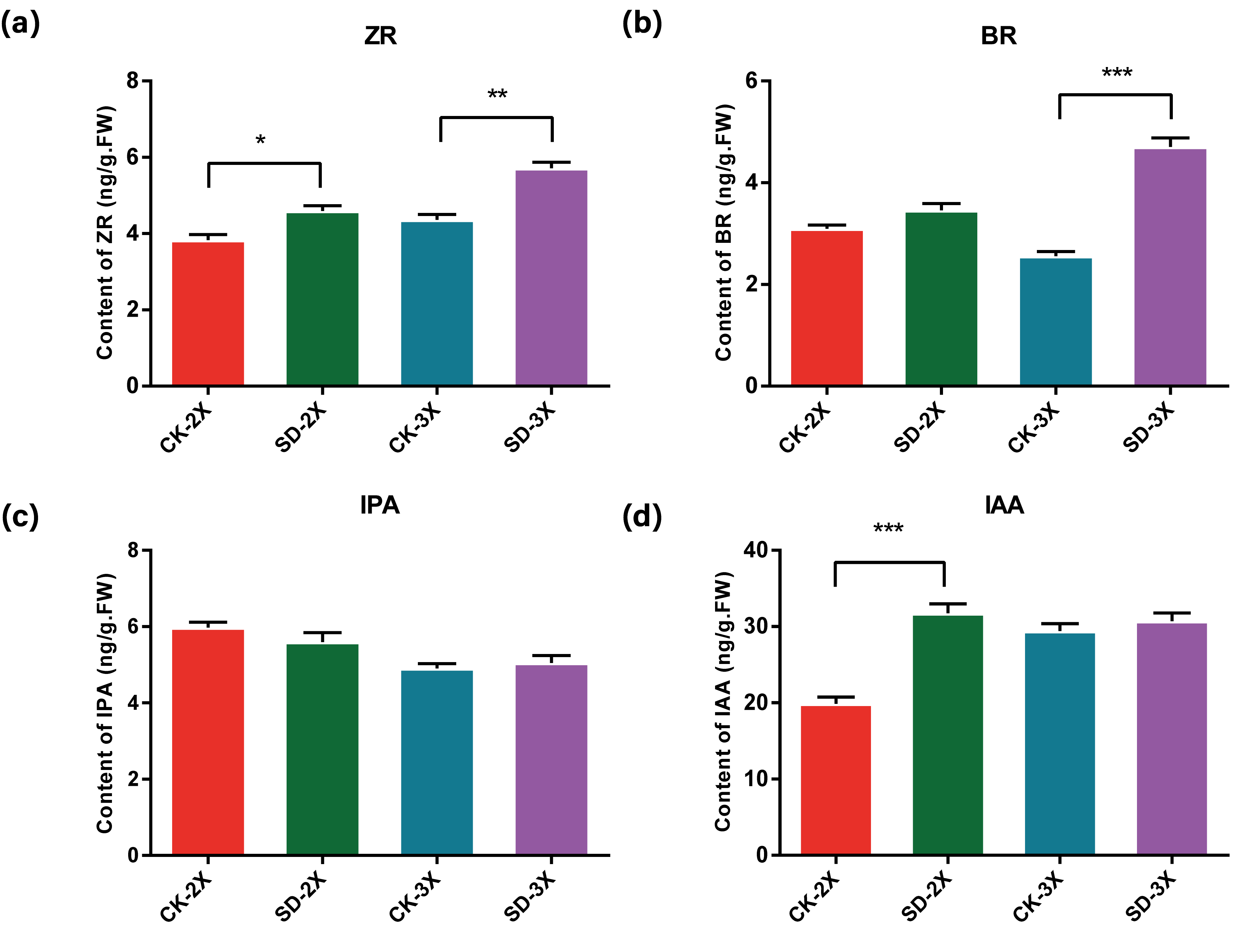

Supplement: Supplementary Figure 2 — Hormonal content in leaves of diploid (2X) and triploid (3X) with 9 days of drought stress for ZR (A), BR (B), IPA (C), and IAA (D). All assays were carried out at least three times and statistical significance levels were calculated using Student's t-test (*P ≤ 0.05; **P ≤ 0.01). CK, control; SD, severe drought. [file Image_2.TIF]

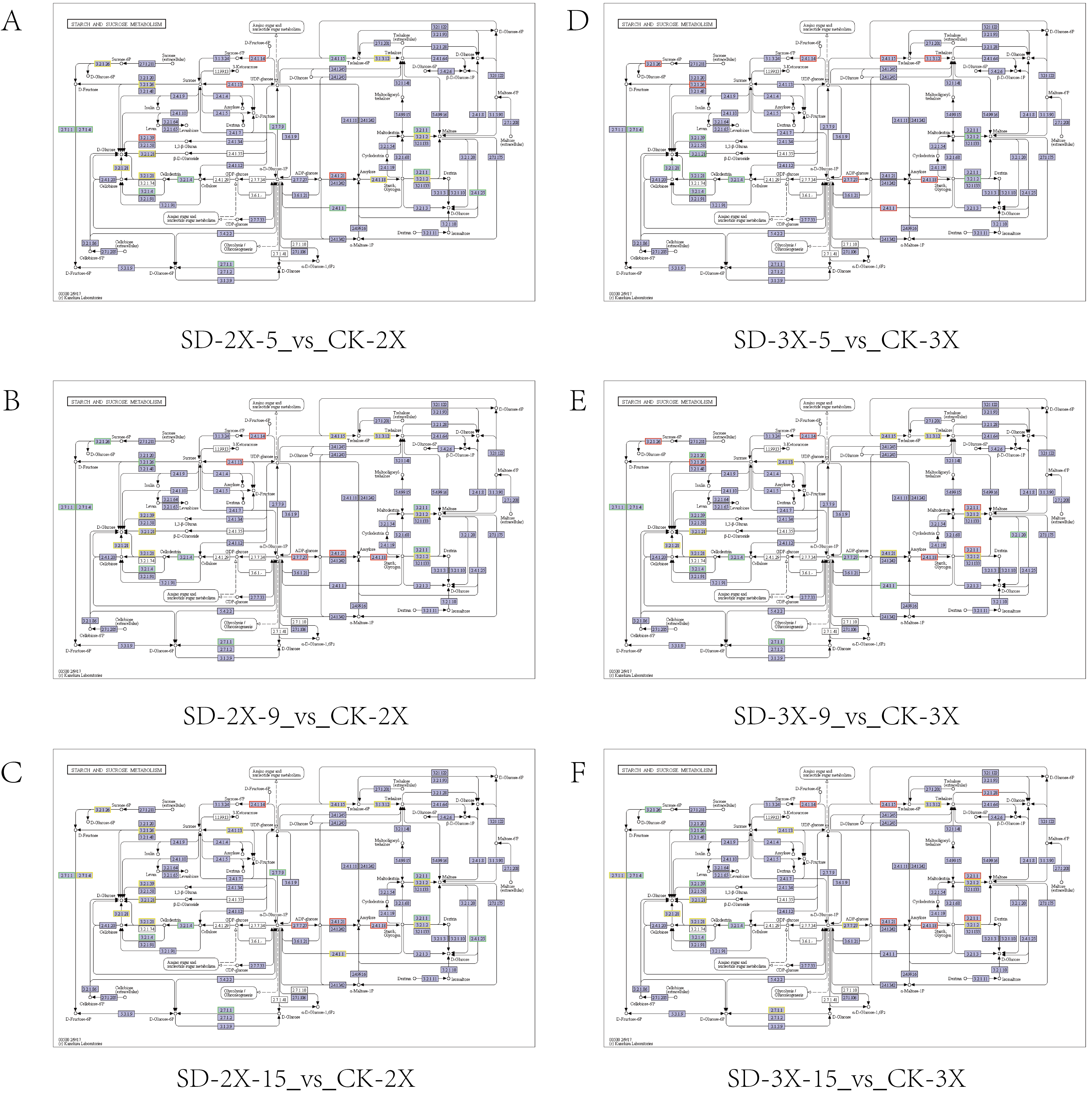

Supplement: Supplementary Figure 3 — Pathways of starch and sucrose metabolism of diploid (2X) and triploid (3X) in response to drought stress. CK, control; SD, severe drought. [file Image_3.TIF]

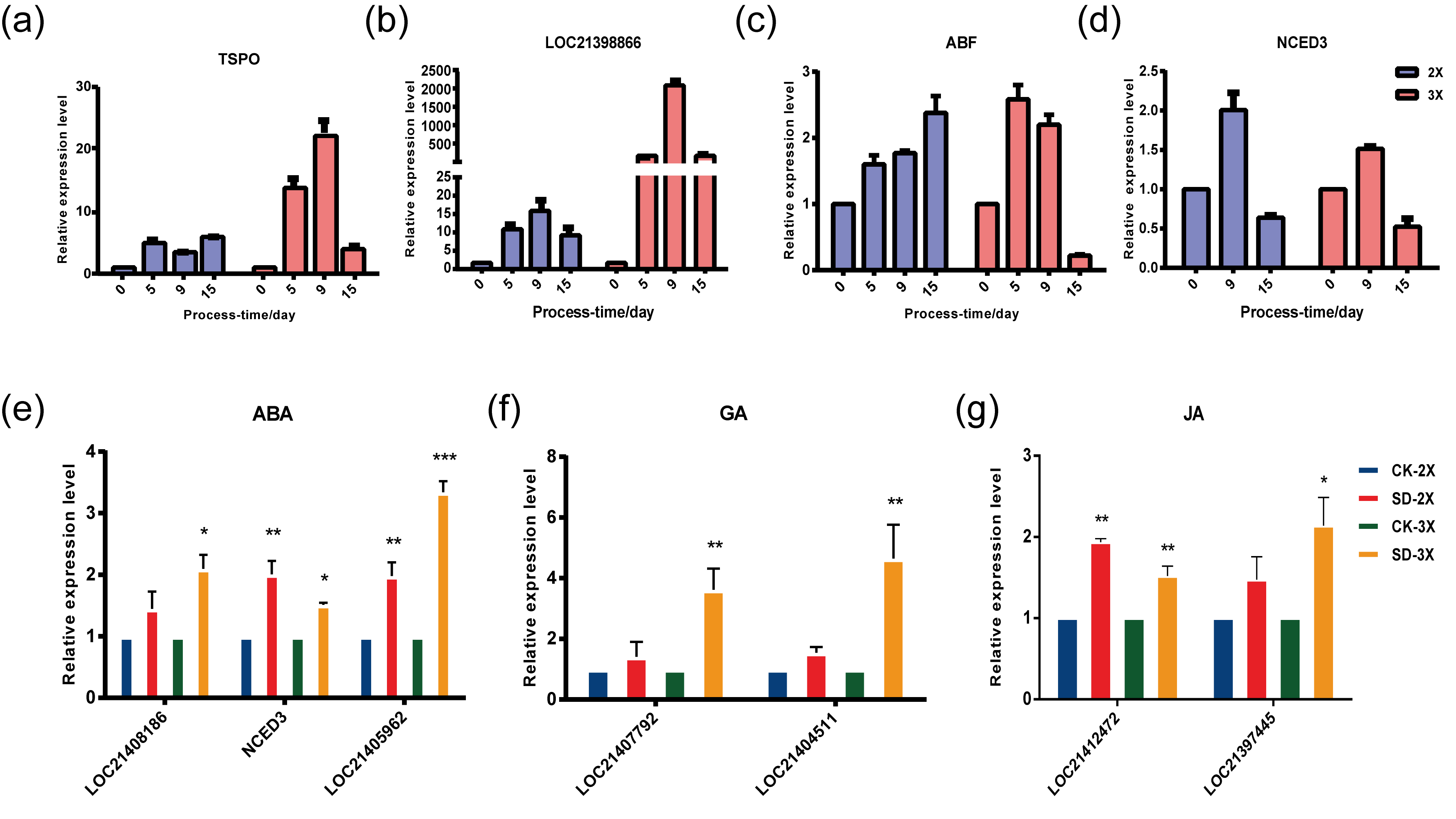

Supplement: Supplementary Figure 4 — Quantitative real-time PCR analysis of genes expression between diploid (2X) and triploid (3X) with drought stress. All assays were carried out at least three times and statistical significance levels were calculated using Student's t-test (*P ≤ 0.05; **p ≤ 0.01; ***p ≤ 0.001). CK, control; SD, severe drought. [file Image_4.TIF]

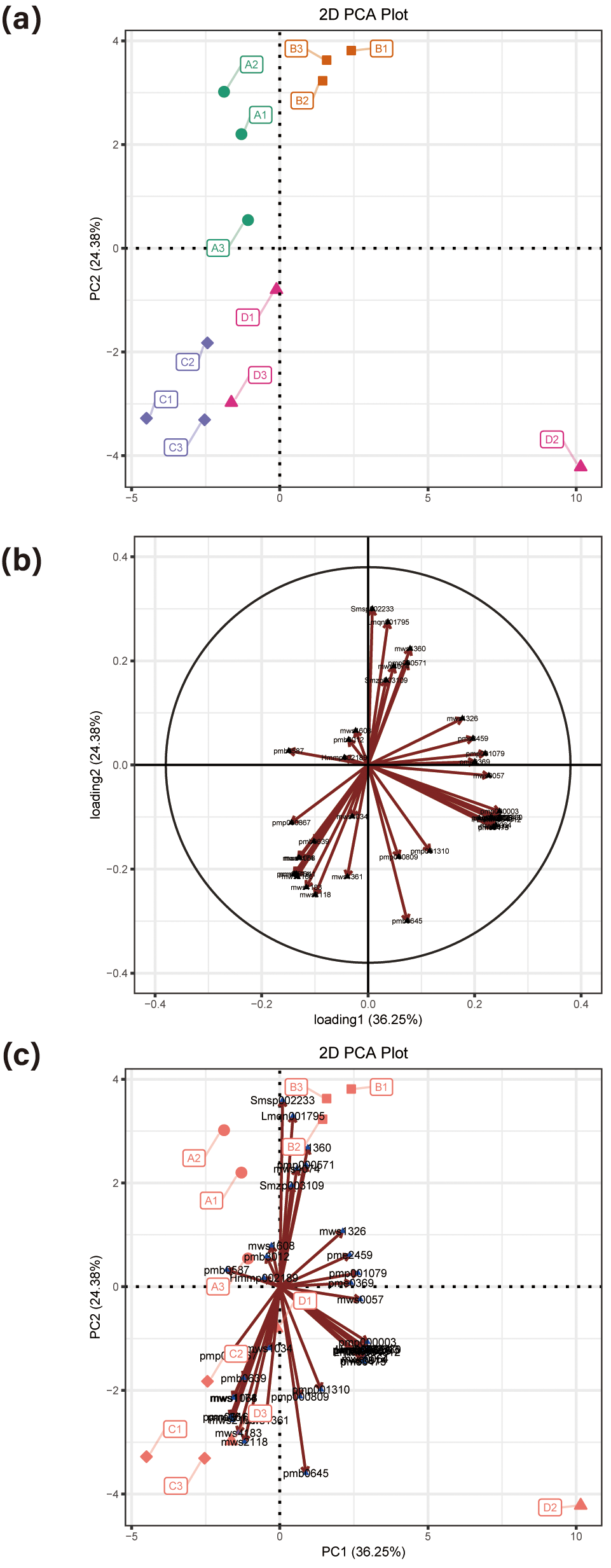

Supplement: Supplementary Figure 5 — Principal component analysis (PCA) of the normalized metabolite intensities in diploid (2X) and triploid (3X). (A) PCA loading plot of PC1 against PC2. Each metabolite is represented by a single dot. (B) PCA plot showing separation by genotype. The accumulation of variance percentage is indicated in each PC. (C) PCA biplot of metabolite and genotype segregation. [file Image_5.TIF]

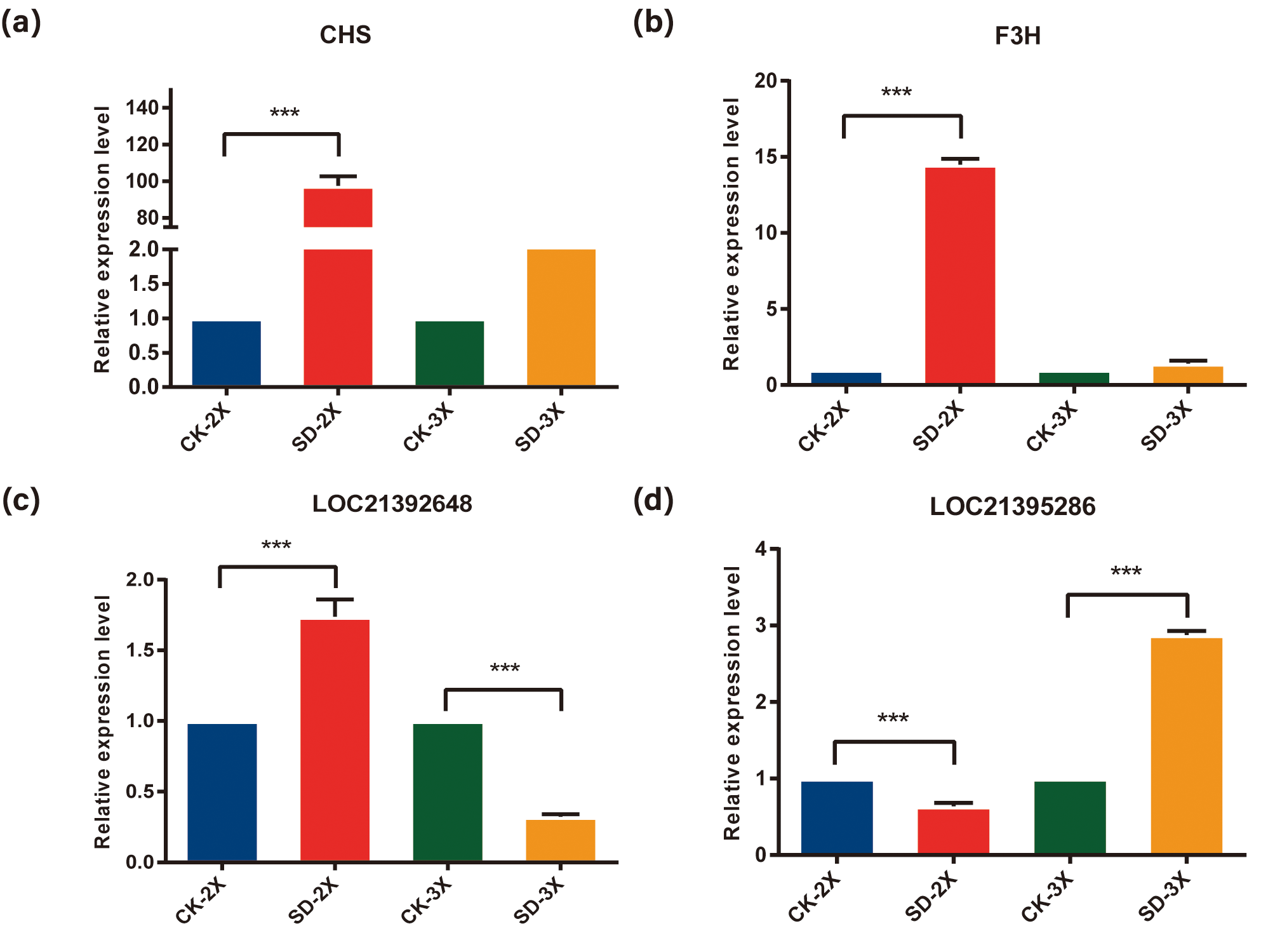

Supplement: Supplementary Figure 6 — Quantitative real-time PCR analysis of LOC21411095 and LOC21396807 expression between diploid (2X) and triploid (3X) with drought stress, which related to flavonoid synthesis. All assays were carried out at least three times and statistical significance levels were calculated using Student's t-test (*P ≤ 0.05; **p ≤ 0.01; ***p ≤ 0.001). CK, control; SD, severe drought. [file Image_6.TIF]

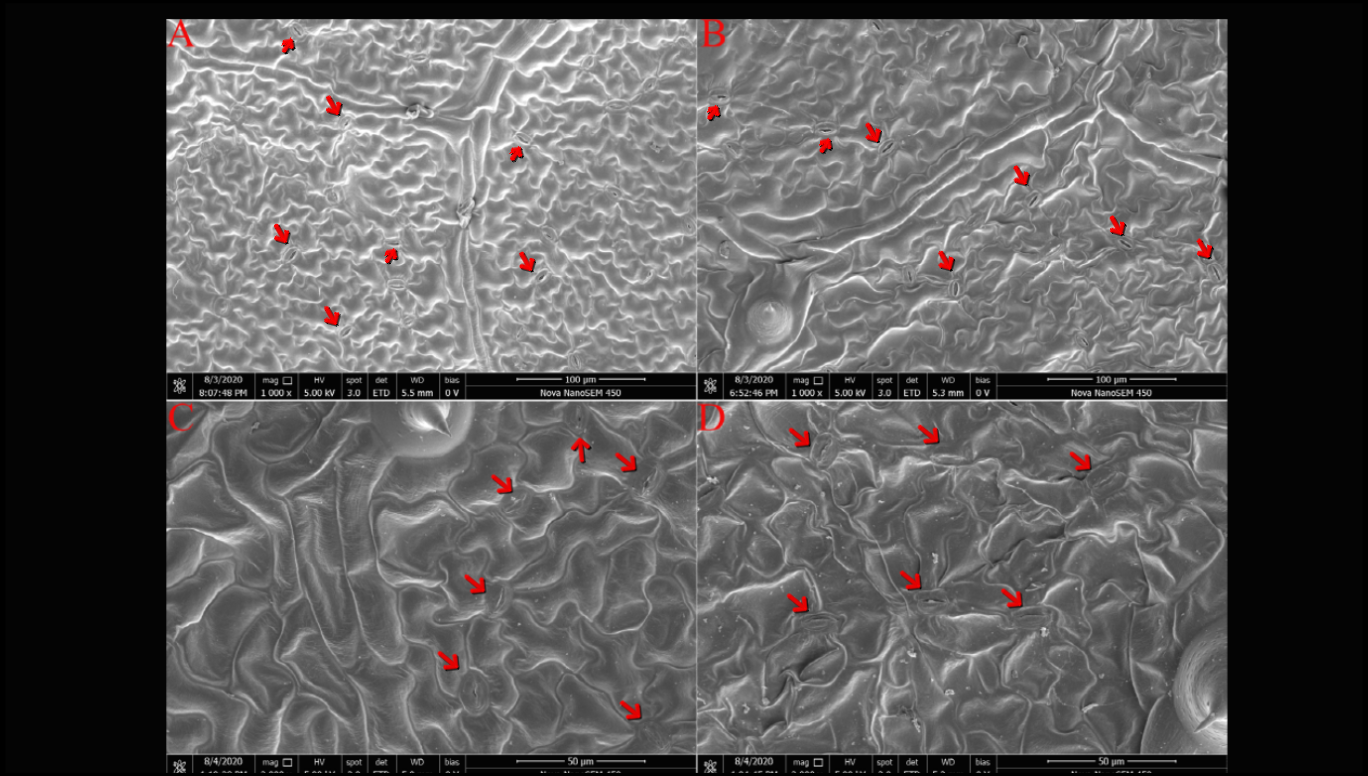

Supplement: Supplementary Figure 7 — Scanning electronic microscopic observation of stomata in diploid(2X) and triploid (3X) leaves. [file Image_7.TIF]
